# A Deep Neural Network Method for Arterial Blood Flow Profile Reconstruction

**DOI:** 10.3390/e23091114

**Published:** 2021-08-27

**Authors:** Dan Yang, Yuchen Wang, Bin Xu, Xu Wang, Yanjun Liu, Tonglei Cheng

**Affiliations:** 1 School of Information Science & Engineering, Northeastern University, Shenyang 110819, China; yangdan@mail.neu.edu.cn (D.Y.); 2070802@stu.neu.edu.cn (Y.W.); wangxu@ise.neu.edu.cn (X.W.); 1800720@stu.neu.edu.cn (Y.L.); 2 Key Laboratory of Infrared Optoelectric Materials and Micro-Nano Devices Liaoning Province, Northeastern University, Shenyang 110819, China; 3 Key Laboratory of Data Analytics and Optimization for Smart Industry, Ministry of Education, Northeastern University, Shenyang 110819, China; 4 School of Computer Science and Engineering, Northeastern University, Shenyang 110169, China; xubin@mail.neu.edu.cn

**Keywords:** arterial blood flow profile reconstruction, artery stenosis, deep neural network, electromagnetic effect

## Abstract

Arterial stenosis will reduce the blood flow to various organs or tissues, causing cardiovascular diseases. Although there are mature diagnostic techniques in clinical practice, they are not suitable for early cardiovascular disease prediction and monitoring due to their high cost and complex operation. In this paper, we studied the electromagnetic effect of arterial blood flow and proposed a method based on the deep neural network for arterial blood flow profile reconstruction. The potential difference and weight matrix are used as inputs to the method, and its output is an estimate of the internal blood flow velocity distribution for arterial blood flow profile reconstruction. Firstly, the weight matrix is input into the convolutional auto-encode (CAE) network to extract its features. Then, the weight matrix features and potential difference are combined to obtain the features of the blood velocity distribution. Finally, the velocity features are reconstructed into blood flow velocity distribution by a convolution neural network (CNN). All data sets are obtained from a model of the carotid artery with different rates of stenosis in a uniform magnetic field by COMSOL. The results show that the average root mean square error of the reconstruction results obtained by the proposed method is 0.0333, and the average correlation coefficient is 0.9721, which is better than the corresponding indicators of the Tikhonov, back propagation (BP) and CNN methods. The simulation results show that the proposed method can achieve high accuracy in blood flow profile reconstruction and is of great significance for the early diagnosis of arterial stenosis and other vessel diseases.

## 1. Introduction

According to investigation [1], cardiovascular diseases such as coronary artery stenosis, coronary heart disease and atherosclerosis have become major diseases that seriously endanger human health. The blood flow velocity in the blood vessel contains physiological and pathological information. Monitoring the changes of blood flow velocity can prevent and control such diseases in advance. Therefore, it is of great value to develop a safe and non-invasive method to monitor blood flow velocity status for the early prevention of common cardiovascular diseases.

Nowadays, common diagnostic methods for arterial stenosis include digital subtraction angiography (DSA) [2], nuclear magnetic resonance angiography (MRA) [3], spiral CT angiography (CTA) [4] and ultrasonic examination [5]. These methods can detect the degree and range of arterial stenosis, but their results depend on the experience of operators, and the operation is complex. Therefore, it is urgent to find a diagnostic device and method that are convenient to use and take into account the advantages of existing technologies. It is well known that blood flow profile information at arterial stenosis can reflect the degree of stenosis, so it is feasible to predict arterial stenosis by measuring blood flow velocity. Because blood is electrically conductive, its flow velocity can be measured by the electromagnetic effect of blood flow theoretically. The fluid detection method based on the electromagnetic effect has been widely used in industrial fields such as wastewater monitoring [6], gas–water two-phase flow measurement [7] and groundwater flow measurement [8]. Additionally, this method has been introduced into the blood flow detection in the medical field in recent years. Maythem [9] proposed a blood flow measurement method based on an electromagnetic flow meter, which verified that the flow potential and blood flow satisfied an approximate linear relationship. Yang et al. [10] studied the numerical simulation about blood flow volume reconstruction based on the weight function theory and proposed the potential application of electromagnetic sensors for predicting the arterial stenosis rate. In 2019, Marinova et al. [11] proposed a multi-electrode electromagnetic flow detection method for non-invasive blood vessel measurement by establishing a three-dimensional (3D) electromagnetic finite element model of the leg. The development of electromagnetic measurement makes it possible to conveniently measure the blood flow velocity of a human based on electromagnetic induction and to monitor vascular lesions. 

Limited by the number of measuring electrodes, the reconstruction of blood flow velocity based on electromagnetic induction is a highly nonlinear and ill-posed inverse problem. Many image reconstruction methods for nonlinear and ill-posed inverse problems have been proposed, which can be divided into traditional algorithms and deep neural networks (DNNs). The traditional algorithms mainly include the Gauss–Newton algorithm [12], the Landweber iterative algorithm [13] and the Tikhonov regularization algorithm [14]. Zhang et al. [15] proposed a combination of algebra reconstruction technique (ART) and total variation (TV) for the image reconstruction of diffuse correlation imaging (DCT). Sun et al. [16] used an improved Tikhonov algorithm for lung cancer monitoring in electrical impedance tomography (EIT). Although these methods are simple and fast, they are easy to obtain through locally optimal solutions. This leads to the relatively low image accuracy reconstructed by traditional algorithms, and it is not enough to meet the requirements of practical medical applications.

Besides, DNNs have gained wide popularity in the problem of ill-posed medical image reconstruction. The application of DNNs in image reconstruction is divided into two parts: some methods combine other reconstruction algorithms with DNNs, and the DNNs are as a post-processing method to improve the image quality. Martin et al. [17] proposed a reconstruction method for 3D EIT. It acquired a linear distribution from the voltage signal through the Gauss–Newton (GN) algorithm and used an artificial neural network (ANN) as a post-processing method to correct the conductivity distribution of the measured area. The phantom and lung data in the experimental results showed that this method can reduce the influence of noise in the measured data and produced high-quality images from a noisy environment. Hamilton et al. [18] coupled the D-bar reconstruction method with a convolution neural network (CNN). Their results showed that CNN can effectively enhance the resulting image of EIT as a method of post-processing D-BAR images. Ren et al. [19] designed a two-stage deep learning method for robust shape reconstruction with EIT. In this method, the rough reconstruction results were obtained by preprocessing the voltage measurement signal with the punishment of regularization term. The results of rough reconstruction and the shape of the lung domain are then input to the convolutional neural network (CNN) for post-processing. Their results showed that this method can reconstruct the lung shape accurately and had good robustness. Some others consider DNNs as a way to reconstruct images directly from measured signals. Chen et al. [20] described a new magnetic induction tomography (MIT) reconstruction algorithm with a stacked auto-encoder (SAE) neural network, which can map non-linearly between input and output. The defects of complex calculation and serious artifacts in traditional algorithms was solved. Li et al. [21] designed a neural network model combining SAE and the logistic regression (LR) to ensure reliable image generation in EIT. The model determined the relationship between the voltage measurement and the internal conductivity distribution. The results showed that its imaging effect is good and it has some anti-noise ability. Jaejun Yoo et al. [22] proposed a novel deep learning approach that learns non-linear photon scattering physics and obtained an accurate 3D distribution of optical anomalies. According to the features of voltage data collected in EIT, Li et al. [23] presented a one-dimensional convolutional neural network (1D-CNN) to solve the inverse problem of image reconstruction. Their results demonstrated that the proposed method had better imaging results, especially for the reconstruction of complex geometric distribution. Chen et al. [24] proposed a MITNet technique to solve the MIT reconstruction imaging problem. This method is constructed by a generative adversarial network (GAN) based on CNN. They verified the feasibility of the proposed method on real data sets, and the experimental results showed that the performance of their method is better than that of the existing methods. Wang et al. [25] proposed a radial basis function neural network based on hybrid particle swarm optimization algorithm to reconstruct images in EIT, which improved the imaging accuracy and the robustness to noise. Therefore, DNNs may become an effective method for blood flow velocity inversion based on electromagnetic induction.

In view of the shortcomings of existing reconstruction algorithms, this paper proposed a novel method for arterial blood flow profile reconstruction based on DNN, integrated CAE and CNN. The CAE takes the weight matrix as input and extracts its low-dimensional features to provide more details in the potential difference reconstruction information. The features of the weight matrix are used to process the reconstructed information from the voltage domain to the velocity domain. In this process, the features of blood velocity distribution are acquired, which are the result of an increase in the dimension of reconstruction information data. The blood velocity distribution features are input into the CNN network and mapped into the blood flow velocity for reconstructing the arterial blood flow profile image. The proposed method was trained and tested by the data obtained from a 2D model of the carotid artery with various stenoses. Correlation coefficient (CC) and root mean square error (RMSE) were adopted as evaluation indexes. Additionally, the effectiveness and robustness of the proposed model are verified. The experiment results show that the method can solve ill-posed image reconstruction for blood flow velocity distribution. Compared to the traditional algorithms and neural network methods, the results demonstrate that the performance of the proposed method is superior to existing methods and shows that it is competitive.

The contributions of this work can be elaborated as follows:

(1) We proposed an arterial blood flow profile reconstruction method based on DNN, with CAE and CNN.
(2) To improve the accuracy of reconstruction image, a data dimension increase strategy is used by CAE and data domain transformation.
(3) A 2D model of the carotid artery with various stenoses was established in COMSOL, and data set was obtained from this model for improving the generalization ability of the proposed method.

The rest of this paper is organized as follows: Section 2 presents the principle of blood velocity reconstruction based on electromagnetic induction and the details of the proposed method; Section 3 describes the generation of data set and the experiment; Section 4 presents the experimental results; Section 5 analyzes and discusses the experimental results; Section 6 summarizes the paper and draws our conclusions.

## 2. Principles and Method

### 2.1. Electromagnetic Effects of Blood Flow

**Figure 1:**
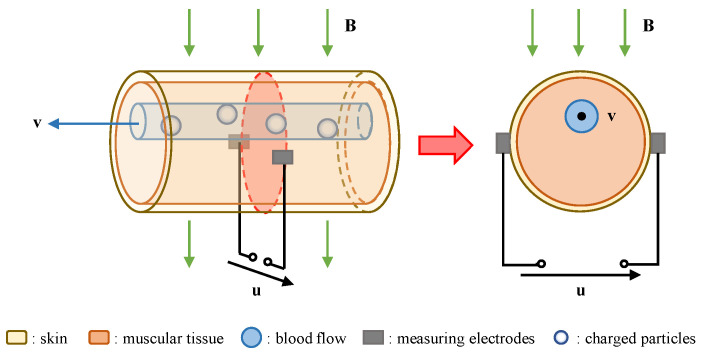
The schematic diagram of induced potential.

Blood flow has both fluidity and electrical conductivity. According to Faraday’s law of induction, when blood flow moves through a uniform magnetic field B with a velocity v as shown in Figure 1, the charged particles of the blood flow will be polarized and offset by the Lorentz force. As charges of opposite polarity accumulate on both sides of the blood vessel, an additional electric field E will be generated.

The electric current density J in blood flow, in the presence of electric and magnetic fields, is given by Ohm’s law:

(1) J=σ(E+v×B)

where σ is blood conductivity and the expression (v×B) represents the local induced electric field caused by the interaction of blood flow and the static magnetic field.

According to the related theory of the electromagnetic field, the scalar potential u is introduced:

(2) ∇·J=0

(3) E=−∇u

Assuming that the conductivity of blood flow is uniform, a general partial differential equation (Poisson’s equation) can be derived. It represents the relationship between the velocity of blood flow with uniform conductivity and the potential:

(4) $$\nabla^{2}u=\nabla \cdot \left(v\times B\right)$$

From (4), the interaction between blood flow velocity and external magnetic field will form an induced potential field within a certain range of the human body, which is the electromagnetic effect of blood flow. The electrical potential distribution of blood flow in B can be solved by using the appropriate boundary conditions. Hence, the potential signal caused by blood flow can be obtained through the measuring electrodes on the skin surface and used to reconstruct blood flow information.

### 2.2. Blood Flow Profile Reconstruction

The Rayleigh–Carson reciprocity theorem is often used to describe the relationship between two independent groups of sources and fields. In the previous work [26], we derived the integral Equation (5) based on the reciprocity theorem, which was used to describe the relationship between the flow inducted potential difference Δu, blood flow velocity v(x,y), and uniform magnetic field B:

(5) $$\Delta u=\iint_{S}v\left(x,y\right)\cdot \left(J_{A}\left(x,y\right)\times B\right)dxdy$$

where, v(x,y) represents the velocity value at each axial point with coordinates (x,y), and $J_{A}\left(x,y\right)$ is the surface current density in the reciprocal field. Further, $J_{A}\left(x,y\right)\times B$ can be regarded as the reciprocal Lorentz force of each axial point f(x,y) generated by the interaction between the reciprocal current and the static magnetic field.

The entire measured section is divided into n small units. Since the radius of the units is small, the information of the center of units can be approximately expressed as the average information of the entire units, and (5) can be further simplified as:

(6) $$\Delta u=\overset{n}{\underset{i=1}{\sum }}f_{i}\left(x,y\right)\cdot v_{i}\left(x,y\right)\cdot S_{i}$$

where n is the number of units, $f_{i}\left(x,y\right)$ is the reciprocal Lorentz force of the i th unit, $v_{i}\left(x,y\right)$ is the velocity value of the i th unit, and $S_{i}$ is the cross-section area of the i th unit.

In practical measurement, many pairs of electrodes are often set on the skin surface. When the measuring electrode pair changes, the reciprocal Lorentz force will also change, so that different potential difference data can be obtained. On the other hand, under the same pair of measuring electrodes, different units correspond to different Lorentz force densities. Through the above analysis, (6), for calculating a single voltage value, is converted into a non-homogeneous linear system of equations applicable to multiple voltages:

(7) $$\left[\begin{array}{c} \Delta u_{1}\\ \begin{array}{c} \Delta u_{2}\\ \begin{array}{c} \vdots \\ \Delta u_{m} \end{array} \end{array} \end{array}\right]=\left[\begin{array}{cc} \begin{array}{cc} f_{11} & f_{12}\\ f_{21} & f_{22} \end{array} & \begin{array}{cc} \cdots & f_{1n}\\ \cdots & f_{2n} \end{array}\\ \begin{array}{cc} \vdots & \vdots \\ f_{m1} & f_{m2} \end{array} & \begin{array}{cc} \ddots & \vdots \\ \cdots & f_{in} \end{array} \end{array}\right]\left[\begin{array}{cc} \begin{array}{cc} S_{1} & 0\\ 0 & S_{2} \end{array} & \begin{array}{cc} \cdots & 0\\ \ddots & \vdots \end{array}\\ \begin{array}{cc} \vdots & \vdots \\ 0 & \cdots \end{array} & \begin{array}{cc} \ddots & 0\\ 0 & S_{n} \end{array} \end{array}\right]\left[\begin{array}{c} v_{1}\\ \begin{array}{c} v_{2}\\ \begin{array}{c} \vdots \\ v_{n} \end{array} \end{array} \end{array}\right]$$

or:

(8) U=[FS]v=Wv

where U is a column vector containing m potential difference data, F is a coefficient matrix containing m×n reciprocal Lorentz force values, S is a diagonal matrix containing n reconstructed units, and v is a column vector containing n velocity values of reconstructed units. We consider [FS] as the m×n weight matrix W, which represents the contribution of flow points at different positions to the potential signal generated by electromagnetic induction.

Therefore, the blood flow velocity can be calculated by the potential difference data U and weight matrix W. Restricted by the number of electrodes, the number of reconstruction units is much more than the amount of potential difference data. So, the problem of artery blood flow profile reconstruction based on the electromagnetic effect is transformed into the problem of solving the underdetermined equation (Equation (8)). The traditional algorithm has a simple computing format and fast imaging speed for solving underdetermined equations. However, they are easy to acquire with locally optimal solutions and do not overcome the underdetermination and ill-posedness of the equation itself fundamentally.

### 2.3. Deep Neural Network-Based Reconstruction

#### 2.3.1. Method Overview

**Figure 2:**
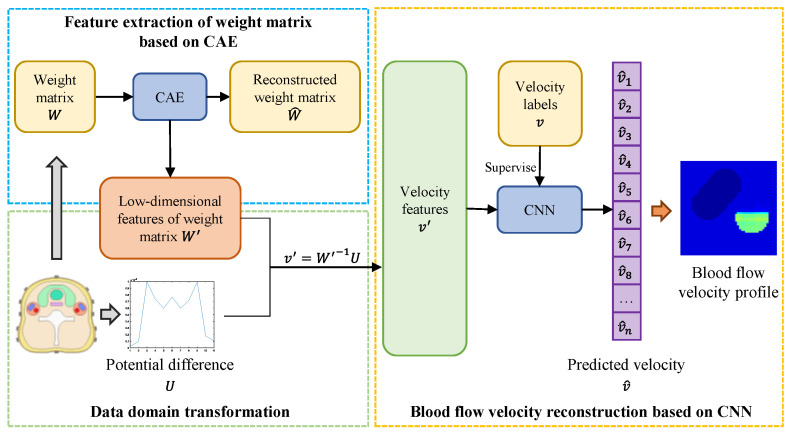
Schematic of the proposed CAE-CNN reconstruction method.

Electromagnetic effect-based arterial blood flow profile reconstruction is a nonlinear ill-posed inverse problem. The ultimate goal of this work is to establish a neural network method for reconstructing the velocity distribution of the arterial blood flow profile, where the input of the method is weight matrix and a set of potential difference values from a 2D carotid artery stenosis model with various stenoses. The output is images of blood velocity distribution as a diagnosis prediction of whether the patient has arteriostenosis or not. Figure 2 shows an overall framework of the proposed method followed by the deep neural network.

Different from other electromagnetic blood velocity reconstruction approaches, the proposed method is mainly divided into three parts. They are feature extraction of the weight matrix based on the convolutional auto-encoder (CAE) network, data domain transformation and blood flow velocity reconstruction based on the convolutional neural network (CNN). Additionally, this method is called the CAE-CNN reconstruction method. The CAE is designed to extract the low-dimensional features $W^{\prime }$ of the m×n weight matrix W for providing more details of reconstruction information. The data domain transformation part recovers the blood flow velocity feature $v^{\prime }$ from the measured potential difference U and $W^{\prime }$. Additionally, with the supervision of the velocity label v, the following CNN maps the blood flow velocity feature $v^{\prime }$ to the predicted blood flow velocity $\hat{v}$.

#### 2.3.2. Feature Extraction of Weight Matrix Based on CAE

**Figure 3:**
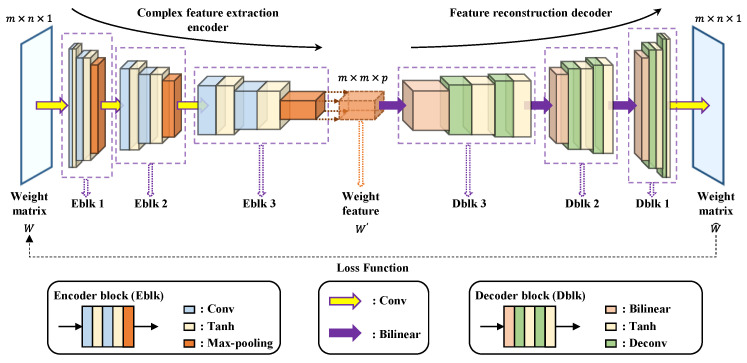
The structure of proposed multi-layer CAE.

CAE is a network for unsupervised feature learning proposed by Masci et al. in 2011 [27]. It combines the advantages and structures of auto-encoder (AE) and CNN. The unsupervised AE can encode the input sample into a low-dimensional representation, while CNN is capable of quickly extracting meaningful features from the input sample [28]. The simple CAE network is shallow, which may make it difficult to extract more complex features. A multi-layer CAE network is proposed to extract the features of the weight matrix W more comprehensively. The proposed multi-layer CAE is consisted of a complex feature extraction encoder and a feature reconstruction decoder. The architecture of our CAE is as shown in Figure 3.

The complex feature extraction encoder involves the application of three blocks denoted with EBlk 1 to 3. The structure of each EBlk contains two convolutional layers and a pooling layer connected in series. The input of each convolutional layer is convolved with the convolution kernels moving according to the given strides to generate the feature map. A hyperbolic tangent (Tanh) function $\zeta_{E}\left(\cdot \right)$ is subsequently added to each convolution layer to activate the convolved features. Additionally, at each convolution step, we increase the number of filters. The last layer of each EBlk is the max-pooling layer to calculate the maximum value of every region with the same shape as the pooling kernel in each feature map. The three EBlks are connected in sequence, and the output of the previous EBlk serves as the input of the next EBlk. The input of CAE is the weight matrix W with the shape of m×n (m<n), and it is calculated correspondingly as per the order of each layer in the encoder. For the sake of simplicity, the feature map in the next layer is generally calculated by the following equation:

(9) $$h_{t}^{k}=\zeta_{E}\left(f_{t}^{k}*h_{t-1}^{k}+b_{1t}^{k}\right)$$

with 1≤t≤6. $f_{t}^{k}$ is the weights of the convolutional kernel, ∗ denotes the 2D convolution, $h_{t-1}^{k}$ denotes the feature map for the $k^{th}$ filter within the (t−1) th layer, and $b_{1t}^{k}$ is the bias term. Each layer is a higher-level abstraction of the previous layer. Therefore, the output of the last EBlk contains the high-level structure and representative information of the original input data, which is the low-dimensional representation of W.

The other purpose of the encoder component is to generate meaningful feature maps of size m×m×p. The size of the kernels (convolution kernels and pooling kernels) and the number of filters are adjusted to determine the shape of the output data. In particular, due to the size of the input W, the size of the kernels is set to the form $1\times l_{conv}$ to ensure that the height of the final output of the encoder remains the same as m, while the width is changed from n to m. Additionally, p is determined by the number of filters in the last convolution layer of the encoder. This means that through the kernel and filter processing of each layer, the size of the data will change correspondingly in the calculation, and the encoder will finally output the feature maps with the specified size. The specific structure and parameters of the complex feature extraction encoder are shown in Table 1.

The data in the table are from the specific data size used in the experiment. In the feature reconstruction decoder part, blocks are reused three times, denoted with DBlk 1 to 3, respectively. Each DBlk is connected in turn, and a convolution layer is added as the last layer of the decoder. The progression of DBlks is the process of upsampling. A bilinear interpolation layer and two deconvolutional layers are arranged sequentially in each DBlk. The bilinear interpolation performs linear interpolation operations on the feature data in two directions severally to enlarge the size of data. Two deconvolutional layers map low-dimensional data to a high-dimensional space by deconvolution calculation. In addition, the number of feature channels is reduced while deconvolutional is performed. Each deconvolutional layer is followed by an activation function to increase nonlinearity. Additionally, the definition of the feature map ${\tilde{h}}_{t}^{k}$ in the t th deconvolutional layer is as follows:

(10) $${\tilde{h}}_{t}^{k}=\zeta_{D}\left({\tilde{f}}_{t}^{k}*{\tilde{h}}_{t-1}^{k}+b_{2t}^{k}\right)$$

where $\zeta_{D}\left(\cdot \right)$ is the Tanh function, ${\tilde{f}}_{t}^{k}$ is the weights of the deconvolutional kernel, and $b_{2t}^{k}$ is the bias term in the deconvolutional layer. The last layer of the decoder is a 1×1 convolution layer, which is mainly used to ensure that the reconstructed output data have the same number of filters as the raw data. Therefore, the final output of CAE $\hat{W}$ can be obtained as follows:

(11) $$\hat{W}=\zeta_{D}\left(H*w_{\mathrm{conv}}+b_{\mathrm{conv}}\right)$$

where H is the map of the last deconvolutional layer, and $w_{\mathrm{conv}}$ and $b_{\mathrm{conv}}$ denote the weights and bias in the last convolutional layer, respectively.

For the decoder, its main function is to restore the m×m×p feature map to the reconstruction of m×n×1 input. The structures of DBlk 1 to 3 and EBlk 1 to 3 are symmetrically distributed. They have the same number of layers, and their operation effects are opposite. The parameters of bilinear interpolation layers are thus set for restoring the data size to the size of the input data in their corresponding pooled layers. The size of the deconvolution kernel and the number of filters are the same as those of the corresponding convolution layers. The specific structure and parameters of the feature reconstruction decoder are shown in Table 2.

Moreover, for a stable gradient and a more robust solution, mean absolute error (MAE) is adopted as the loss function in our CAE.

(12) $$Loss\left(W,\hat{W}\right)=\frac{1}{n}\sum_{i=1}^{n}\left|W_{i}-{\hat{W}}_{i}\right|$$

where $W_{i}-{\hat{W}}_{i}$ is the reconstruction error of the weight matrix with CAE.

The solver adopts the adaptive moment estimation (ADAM) algorithm to update the network weights instead of the classical stochastic gradient descent algorithm. The ADAM algorithm uses the momentum and adaptive learning rate to speed up the network convergence with good robustness [29].

When the CAE network is well trained, the complex feature extraction encoder will provide a low-dimensional representation of the input weight matrix W, known as the weight feature matrix $W^{\prime }$. Additionally, the feature reconstruction decoder is deployed to evaluate the reliability of the CAE-based weight feature matrix $W^{\prime }$.

#### 2.3.3. Data Domain Transformation

After the weight feature matrix $W^{\prime }$ is extracted by the unsupervised learning of CAE, the m×1 potential difference data U are transformed from the voltage domain to the blood velocity domain by combining them. The data domain transformation process is divided into two steps:

Firstly, solving the inverse matrix of $W^{\prime }$. $W^{\prime }$ is a three-dimensional matrix with the shape of m×m×p, which can be regarded as composed of p sub-feature matrices $W_{sub}$s with the shape of m×m. The $W_{sub}$ is an m-order square matrix, so its inverse matrix ${W_{sub}}^{-1}$ can be solved directly. The inverse matrix of $W^{\prime }$ is formed by ${W_{sub}}^{-1}$s, and its shape is m×m×p.

**Figure 4:**
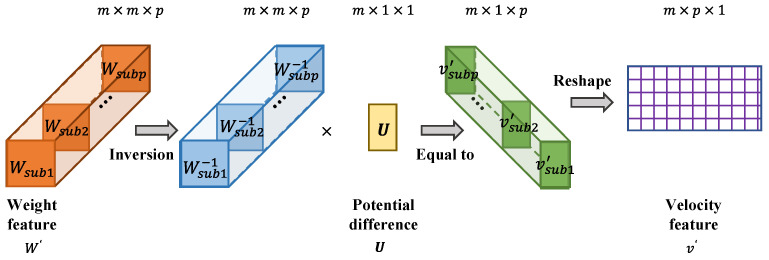
The schematic diagram of the data domain transformation process.

Secondly, on the basis of (8), the potential difference U is processed by the inverse matrix of $W^{\prime }$. Data domain transformation is realized by this process. The m×1 potential difference U is multiplied by each m×m ${W_{sub}}^{-1}$ to obtain the m×1 sub-feature matrix of velocity. The data are mapped from the voltage domain to the velocity domain by the following formula:

(13) $$v_{sub}=W_{sub}^{-1}U$$

where $v_{sub}$ is the m×1 sub-feature matrix of velocity, and the number of $v_{sub}$ is p. These $v_{sub}$s form a m×1×p three-dimensional matrix. The matrix is reshaped to acquire a $v^{\prime }$ of shape m×p. It should be noted that $v^{\prime }$ is not the final velocity distribution but the velocity domain data with the features of velocity distribution. $v^{\prime }$ is treated as the result of the data domain transformation. The procedure of data domain transformation is shown is Figure 4.

#### 2.3.4. Blood Flow Velocity Reconstruction Based on CNN

**Figure 5:**
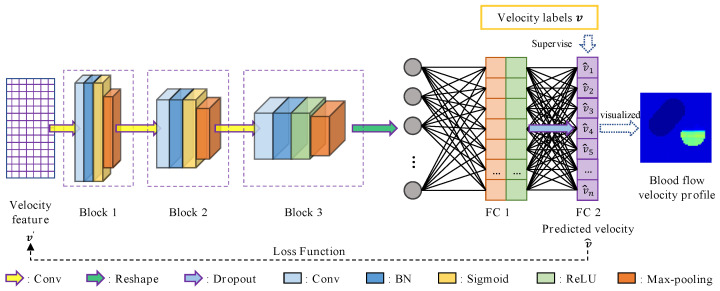
The structure of CNN in blood flow velocity reconstruction.

The CNN in this paper is designed to reconstruct the n×1 blood flow velocity value vector by learning the m×p distribution features $v^{\prime }$ of the flow velocity. The general architecture of our CNN network is shown in Figure 5.

The proposed CNN network mainly incorporates two sequential parts: (1) three reused blocks 1 to 3, whose purpose is to extract different features of $v^{\prime }$; (2) two fully connected layers (FC 1 to 2), which synthesize the features extracted from the previous three blocks.

The blocks 1 to 3 are with the same structure. Each block is constructed by a convolutional layer, a batch normalization (BN) layer, an activation function and a max-pooling layer. Through the convolution operation, it generates new feature maps. The sizes of convolutional kernels and numbers of filters in these convolutional layers are set to 1×31, 1×1, 1×1, and 6, 12, 24, respectively. The BN layer is taken to solve the gradient vanishing problem. It is placed between the convolution layer and the activation function to normalize the output of the convolution layer. This is able to pull the data values mapped by the followed activation function from the saturated region to the unsaturated region. Activation functions in the three blocks are separately the sigmoid function, the sigmoid function and the rectified linear unit (ReLU). The max-pooling layer with a size of $1\times l_{pool}$ form is introduced to calculate the maximum value of every $1\times l_{pool}$ region in each normalized and activated feature map. The shapes of pooling kernels are severally 1×16, 1×9, 1×2.

The output of the block 3 is flattened into a one-dimensional vector, which feeds the last part of the network conformed by fully connected layers. Fully connected layers are defined by their number of node units. Each node unit in the fully connected layer is connected to all the nodes in the previous layer. These units compute a weighted sum of their inputs from the previous layer and pass the result through an activation function. In our CNN network, the number of node units in the two fully connected layers is n. Additionally, FC 1 passes the result through ReLU, while the activation function is not set in FC 2. The final output of the CNN network is the result of passing through FC 2. Furthermore, a dropout layer is placed between FC 1 and FC 2 to drop a fraction of the FC 1 nodes randomly at every iteration with a probability of 0.5. Its objective is to prevent overfitting. The specific structure and parameters of our CNN are shown in Table 3.

The loss function and solver of our CNN is the MAE function and ADAM algorithm, respectively. The flow velocity feature $v^{\prime }$ is input to CNN, and the real flow velocity values v are labels for supervised training. The training was carried out through the ADAM optimizer in order to find the most suitable weights and bias for the model, which could result in less MAE loss. The network finally outputs the n×1 predicted flow velocity values $\hat{v}$, and they are visualized to realize the image reconstruction of blood flow velocity distribution in the measured section.

## 3. Experiments

### 3.1. Data Set

A reliable and sufficient data set is the necessary condition for training and optimizing the deep learning network to test the effectiveness of the proposed method. Because it is difficult to obtain true blood flow velocity distributions in arterial profile as the data set, a 2D carotid artery stenosis model with different rates of stenosis is generated from the fascia layer cross-section of the human neck [30]. The course of carotid artery stenosis model establishment is described in detail in Section 3.1.1. The data set acquisition process is described in Section 3.1.2.

#### 3.1.1. Model Establishment

**Figure 6:**
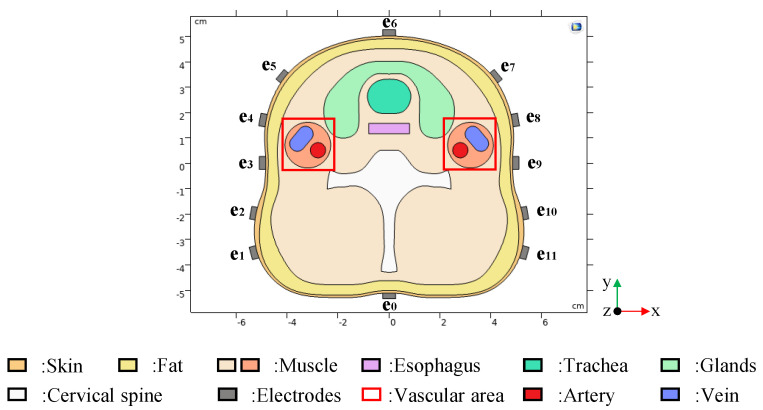
A 2D carotid artery stenosis model with different rates of stenosis.

The fascia layer cross-section of the human neck shows the basic components of the carotid internal, including skin, fat, muscle, cervical spine, esophagus, glands, trachea, artery and vein. The edge coordinates of each biological tissue in the fascia layer cross-section are extracted. According to these coordinates, the 2D carotid artery stenosis model, as shown in Figure 6, was constructed in COMSOL Multiphysics by interpolation curve technology. The internal structure of the human neck is basically restored in the model. The model includes electrodes in addition to the structure of the human neck. A total of 12 electrodes (e0, e1 … e11) are used to detect potential differences in the surface of the skin. e0 is the grounded reference electrode and (e1 … e11) are the measuring electrodes.

The real physical parameters of each organization were set after the geometric modeling was completed. Referring to the relevant biomedical literature [31,32], the typical conductivity values, as shown in Table 4, are assigned to each tissue.

Moreover, the normal carotid artery adopts the periodic pulsating blood flow velocity according to the real physiological value [33]. The variation cycle of arterial blood flow velocity is generally about 0.8 s, and it is affected by the cardiac cycle. When the artery stenosis occurs, the blood velocity in the vessel will change accordingly [34,35]. So, the relationship between arterial stenosis rate and blood flow velocity needs to be analyzed.

**Figure 7:**
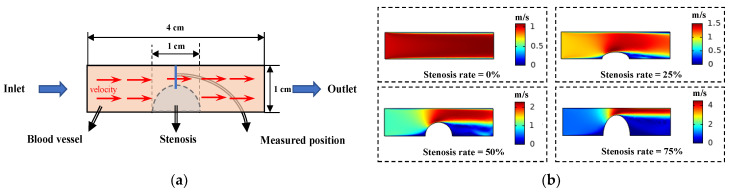
The relationship between blood flow velocity and vascular stenosis rate was established: (**a**) the simple blood vessel model with stenosis; (**b**) the distribution of the intravascular flow velocity at different stenosis rates.

A simple blood vessel model is constructed in COMSOL, as shown in Figure 7a. The left boundary of the model is the flow velocity inlet, and the right boundary is the flow velocity outlet. The inlet velocity was set to 1 m/s. The distributions of the intravascular flow velocity at different stenosis rates are as shown in Figure 7b when the stenosis degree of the model vessel is changed.

During this process, the levels of blood flow velocity at the stenosis corresponding to different stenosis rates were obtained. Since the inlet flow velocity is set as a unit flow velocity, these velocity values can be regarded as the blood flow velocity variation coefficient of the stenosis at different stenosis rates. The pulsating blood flow velocity multiplied by the coefficient is the arterial blood flow velocity in the model. Additionally, the arterial blood flow is set to flow in the positive direction of the z axis.

The venous blood velocity is relatively slow and stable. It was set at an average of 0.08 m/s. Additionally, the vein flows in the opposite direction to the artery.

#### 3.1.2. Data Set Generation

**Figure 8:**
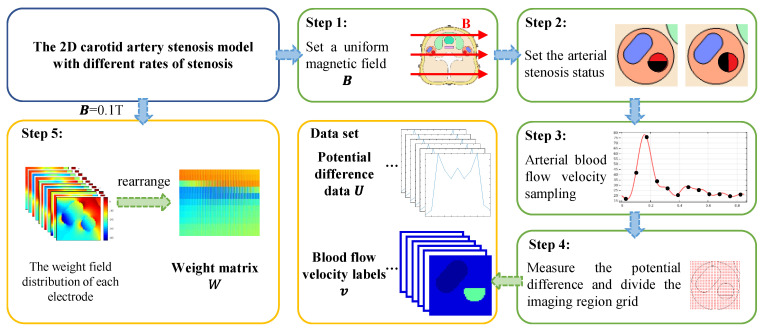
Workflow of the data set generation.

The data set for the training and testing network is generated from the carotid artery stenosis model. The generation process of the data set is shown in Figure 8.

Step 1: The model was placed in an external excitation magnetic field, which was set as a uniform magnetic field in the positive direction of the *x* axis. The field intensity of the uniform magnetic field was set to change from 0.02 to 0.1 T, and the change step was 0.02 T.

Step 2: In each external magnetic excitation environment, the arteriostenosis is simulated by removing a portion of the artery, and the blood flow velocity of the stenosis portion is set to 0 m/s. The stenosis portion occupies 0% (no stenosis) ~90% of the entire artery region. The stenosis rate varies at 10% intervals starting at 0%. Additionally, the stenosis region is expanded along the positive x axis and negative y axis, separately.

Step 3: Under the condition of uniform magnetic field intensity and arterial stenosis rate changing, the arterial blood flow velocity of each case at a specified moment in a pulsation period is set. We take one pulsation period of blood flow velocity fluctuation as 0.85 s. The blood flow velocity is sampled every 0.01 s and the current sampling velocity is assigned to the arterial blood flow. So, the distribution of blood flow velocity corresponding to a different magnetic environment and degree of arterial stenosis is determined.

Step 4: The established model and the velocity distribution are combined to generate the voltage data set. The reference electrode e0 is grounded and the boundary voltage is extracted through the remaining 11 measuring electrodes e1~e11. Blood velocity labels are generated by dividing the measured area into reconstruction units. For the carotid artery stenosis model, the blood flow area accounts for less than 10% of the entire neck cross-section area. The local imaging method is adopted in this paper to reduce unnecessary computation. According to the established model, the positions of the artery and vein are determined, and a rectangular area containing these blood vessels is delimited as the vascular area. The vascular area with arterial stenosis is divided into several grids and the number of reconstruction units of blood flow velocity distribution was 2601. The velocity values of these units are the blood velocity labels. From Step 4, 8170 groups of the voltage data set and velocity labels generated by carotid artery models are acquired. Each sample incorporates a 11×1 voltage value column vector and a 2601×1 blood flow label column vector.

Step 5: The weight matrix is calculated from a standard carotid artery stenosis model without arteriostenosis, in which the uniform magnetic field strength is 0.1 T. The measuring electrodes are energized in turn, and the weight field distribution of each electrode is calculated, respectively. Additionally, they were rearranged into a weight matrix with a shape of 11×2601, as per the corresponding relationship between different electrodes and different reconstruction units.

### 3.2. Network Training

The carotid artery stenosis data set is applied for CAE-CNN network training. The reconstruction process of blood flow velocity distribution based on CAE-CNN is as follows:

Firstly, the weight matrix is trained by CAE. The weight matrix W with the shape of 11×2601 is standardized and then input to the unsupervised network CAE for training. The number of training iterations is 3000, and the learning rate is set as 0.0001. The reconstruction of the weight matrix is compared to the input. The MAE loss function is calculated to quantify the reconstruction error. The error is propagated backwards through the network using the ADAM algorithm to update all the weights involved in the network. When the network training is finished, the weight matrix is compressed by CAE into a weight feature matrix $W^{\prime }$ with the shape of 11×11×60. Additionally, $W^{\prime }$ includes 60 11×11 sub-feature matrices $W_{sub}$s.

Then, the voltage data are mapped onto the velocity domain. The $W_{sub}$s get through the CAE network, and the 11×1 voltage value column vector U of a sample is calculated according to Formula (13), and 60 velocity sub-features $v_{sub}$s with the shape of 11×1 are obtained. These $v_{sub}$s are reshaped into an 11×60 feature matrix $v^{\prime }$ of blood velocity distribution. Additionally, the voltage data of each sample are processed in the same way.

Finally, the blood flow velocity is reconstructed by CNN. The 6536 groups of samples are randomly selected for training, and the remaining 1634 groups of samples are used as the test data. The training set of blood flow velocity feature $v^{\prime }$ is normalized and then input into CNN. The 2601×1 blood flow label column vector v is used as labels for the supervised learning of the network. Additionally, the learning rate is set to 0.00001, the batch-size is 817 and the number of iterations is 1500. The predicted velocity values $\hat{v}$ of 2601 independent subdivision units are output by CNN. After the network training, the imaging quality is tested with randomly assigned test sets, and the reconstructed velocity distribution is calculated and visualized.

A PC equipped with an Inter(R) Core(TM) i5-6200U CPU @ 2.30GHz and a 64-bit system type with 8 GB of memory is used for the implementation of the proposed method. The operating system of the computer is 64-bit Win10. The algorithmic programming, training and testing of CAE and CNN is implemented under Python 3.5 with TensorFlow 1.10.0. The calculation of data domain transformation and results visualization are carried out in MATLAB R2019a. The whole process of the proposed method for training, calculating and predicting results took a total of 12.7 h.

## 4. Results

In order to evaluate the reconstruction ability of the proposed method, three image reconstruction methods, namely, the Tikhonov regularization algorithm, the BP neural network and the CNN network, are compared. They reconstruct the distribution of blood flow velocity directly from the potential difference. For the purpose of highlighting the role of the CAE, the network architecture and training parameters of the CNN in the comparison methods and the CNN part of proposed method are the same. On the other side, several groups of tests are conducted to evaluate the robustness of the CAE-CNN method. The results of the training and testing of the network are as follows.

**Figure 9:**
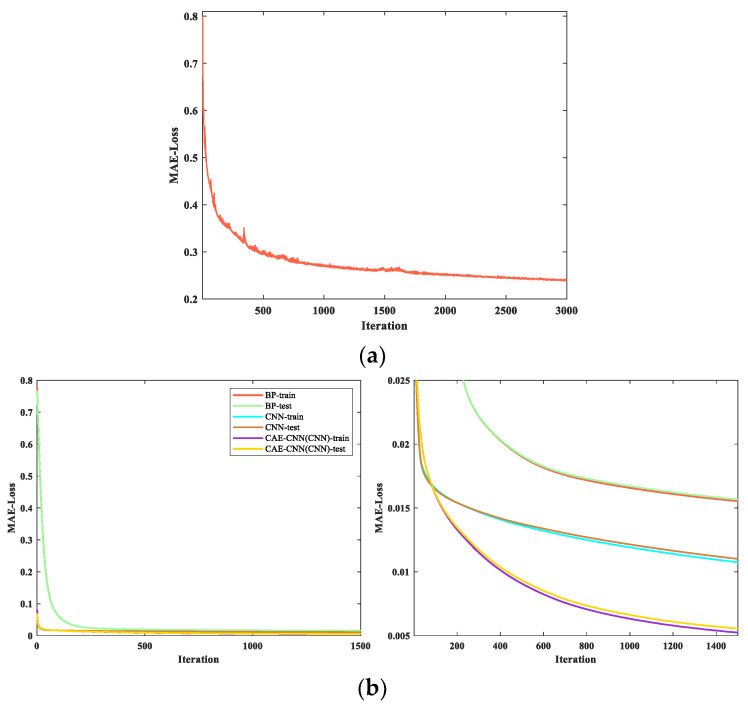
Loss function curve: (**a**) loss function curve of the CAE network when training the weight matrix; (**b**) training and testing curves for different neural network reconstruction methods. The image on the right is a larger version of the one on the left.

In the feature extraction of the weight matrix process, the curve of the loss change during the CAE network training is shown in Figure 9a. In the blood velocity reconstruction part of CAE-CNN, the CNN loss curve in the process of training and testing is shown in Figure 9b. It represents the variation of the loss value of the CAE-CNN method. For evaluating the performance of our proposed method, the training and testing curves of different neural network methods are illustrated in Figure 9b.

In Figure 9a, the loss function curve of CAE tends to go down overall with the increase in iteration times. The decline of the curve is rapid at first and then tends to be gentle, the loss value decreases from 0.801 to 0.240. When the number of iterations is 3000, the loss value still has a downward trend but basically remains unchanged. Considering the factor of training duration, the CAE network is set to stop training when the iteration reaches 3000 generations. As shown in Figure 9b, loss curves of different methods have the consistent trend. The loss curve of CAE-CNN converges more slowly than that of CNN, but its final loss value is the smallest. In the period of testing, the loss can converge from 0.069 to 0.005. All of these indicate that the CAE-CNN network model achieves good performance both in training and testing.

### 4.1. Evaluation Metrics

To quantify the quality of the reconstructed images of the CAE-CNN method, the root mean square error (RMSE) and the correlation coefficient (CC) are taken as the metrics of the network’s performance. The expressions of RMSE and CC are defined as follows:

The sum of the square of the deviations between the predicted and real values for each reconstruction unit is divided by the total number of reconstruction units in a sample, which is used to measure the deviation between the reconstructed blood flow velocity $\hat{v}$ value and the true value v. The RMSE of a sample is calculated as follows:

(14) $$RMSE\left(v,\hat{v}\right)=\sqrt{\frac{1}{n}\sum_{i=1}^{n}{\left(v_{i}-{\hat{v}}_{i}\right)}^{2}}$$

The similarity between the predicted blood flow velocity value and the true value is measured by the correlation coefficient, which is between −1 and 1. The closer the value of CC is to ±1, the more linearly the predicted value is related to the true value. When CC value is 0, it means that the measurement object is linearly independent. The CC of a sample is calculated as follows:

(15) $$CC\left(v,\hat{v}\right)=\frac{\sum_{i=1}^{n}\left({\hat{v}}_{i}-\bar{\hat{v}}\right)\left(v_{i}-\bar{v}\right)}{\sqrt{\sum_{i=1}^{n}{\left({\hat{v}}_{i}-\bar{v}\right)}^{2}}\sqrt{\sum_{i=1}^{n}{\left(v_{i}-\bar{v}\right)}^{2}}}$$

where $\bar{\hat{v}}$ is the average of the predicted blood flow velocity vector $\hat{v}$, and $\bar{v}$ is the average of the true velocity vector v.

### 4.2. Arterial Blood Flow Profile Reconstruction Results

**Figure 10:**
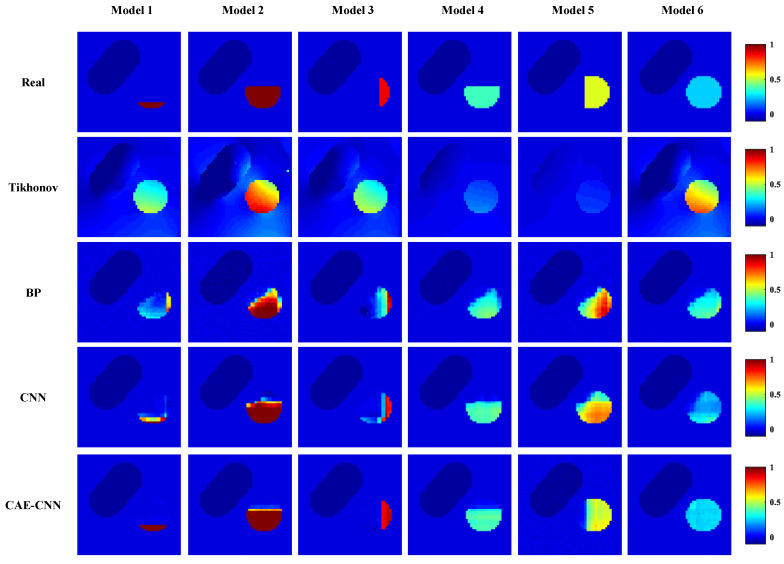
Reconstructions of the blood velocity distribution from noiseless data using different reconstruction methods.

Reconstructed images of blood velocity distribution were obtained by visualizing the predicted blood flow velocity values of a network’s output. Figure 10 shows some of the reconstruction results using different methods. The top of Figure 10 shows the actual blood flow velocity distribution of the carotid artery stenosis model in six cases, while the lower side is the blood flow velocity distribution predicted by the proposed method (row 5) and other image reconstruction methods (row 2 to 4).

In Figure 10, the reconstruction results of CAE-CNN are approximately similar with the target blood flow velocity distribution, which proves the feasibility of the CAE-CNN method for the arterial blood flow profile reconstruction problem. Comparing the prediction results of these groups, the CAE-CNN method can well distinguish the degree and location of arterial stenosis. Moreover, the network can greatly reduce the reconstruction artifacts, and its imaging boundary of the narrow area is relatively clear.

**Figure 11:**
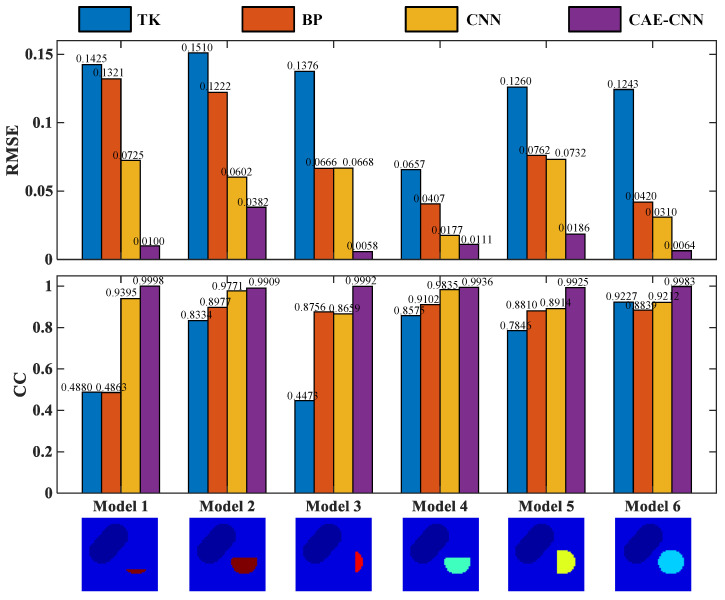
Evaluation index results of image reconstruction examples in Figure 9.

The above six test models are quantitatively evaluated with these two indexes. The RMSE and CC of each model’s reconstruction result in Figure 10 are shown in Figure 11.

From the results recorded in Figure 11, CAE-CNN has the best reconstruction effect on all six samples. The CC values of the reconstructed blood flow velocity distribution of the six models range from 0.9909 to 0.9998, and the RMSE are all less than 0.0382. The results of quantitative evaluation show that the images of blood flow velocity distribution reconstructed by CAE-CNN have good image quality.

For evaluating the CAE-CNN network more comprehensively, the average values of the predicted blood flow velocity distribution results of 817 samples were calculated. The 817 samples are randomly selected from 1634 test sets. The calculation results of evaluation indexes are listed in Table 5. 

In Table 5, the mean values of the RMSE and CC of 817 samples reconstructed by the CAE-CNN method are 0.0333 and 0.9721, respectively. On average, the proposed method achieved 74.96%, 63.25%, and 56.75% (RMSE) and 122.65%, 26.86%, and 16.31% (CC) quantitative metrics improvement compared with the Tikhonov, BP, and CNN methods, respectively. It can be seen from Table 5 that whether it is from the RMSE, which evaluates the accuracy of the blood flow velocity estimating ability, or from the CC, which evaluates the image reconstruction quality of the method, the CAE-CNN network’s results are very good in all quantitative criteria. This shows that the CAE-CNN network can extract more abundant features from limited reconstructed information, not only reflecting the situation of arterial stenosis, but also achieving a high blood velocity estimation accuracy and obtaining higher image reconstruction quality.

### 4.3. Anti-Noise Performance Test Results

To test the anti-noise performance of the proposed method, the measured potential difference signal is contaminated with different degrees of noise. The level of added noise is expressed in (16).

(16) $$SNR=20\mathrm{lg}\frac{U_{s}}{U_{n}}$$

where signal-to-noise ratio (SNR) refers to the ratio of signal to noise in a measurement system, used to evaluate the level of noise, and $U_{s}$ and $U_{n}$ represent the effective value of the signal and noise voltages, respectively. The white Gaussian noise at 60, 50, 40, 30, and 20 dB SNR is added to the potential.

**Figure 12:**
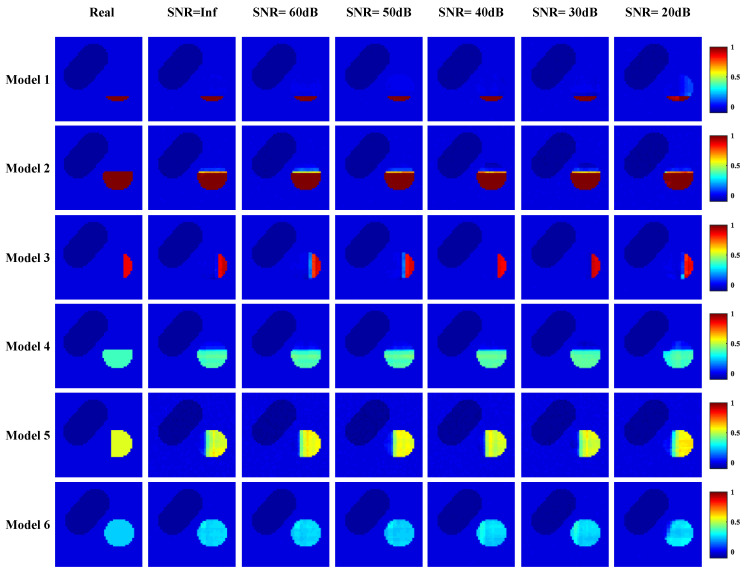
Reconstruction results of CAE-CNN from data with varying levels of noise.

**Figure 13:**
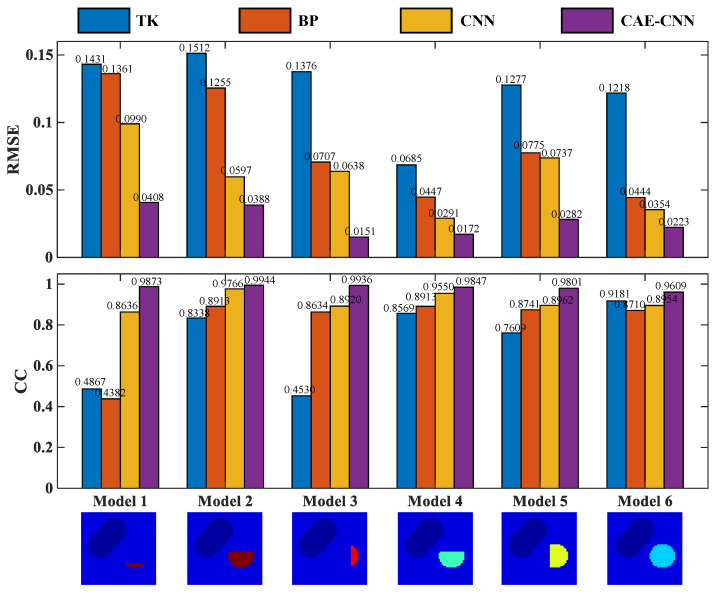
Evaluation index results of image reconstruction examples in Figure 12 when SNR = 20 dB.

Figure 12 shows the reconstruction results of CAE-CNN at different noise levels. With the increase in noise, the blood flow velocity distribution of the proposed method can reflect the arterial stenosis. Additionally, the difference between the reconstruction effect under noise and that without noise is small. The boundary of the reconstructed stenosis area is still clear, and the reconstructed blood flow velocity distribution is close to the real distribution. When SNR = 20 dB, there are few artifacts in CAE-CNN reconstruction images, but this does not affect the judgment of arterial stenosis. The RMSE and CC of each model’s reconstruction result in Figure 12 are listed in Figure 13.

To evaluate the proposed method objectively, Figure 13 shows the evaluation index results of the six models reconstructed by different methods when SNR = 20 dB. RMSE values of the six models reconstructed by the CAE-CNN method are all less than 0.0408, and CC values are all greater than 0.9606.

**Figure 14:**
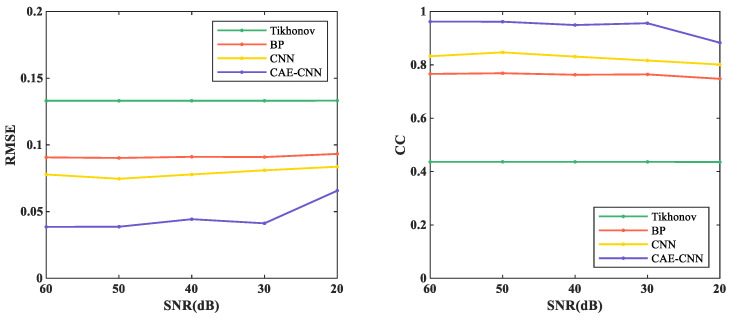
The average results of evaluation indexes for 817 noisy samples.

The quantitative analysis is made from the perspective of evaluation indicators, and the average RMSE and CC of different methods in the random 817 test samples with different noise levels are shown in Figure 14.

From Figure 14, RMSE decreased from more than 0.1331 to no less than 0.0657, and CC increased from less than 0.4358 to more than 0.8830 by using CAE-CNN. As the measurement noise disturbance intensifies, the reconstruction performances of the CAE-CNN network were just slightly decreased. When the SNR is between 60 and 30 dB, the RMSE and CC of CAE-CNN reconstruction results vary from 0.0386 to 0.0443 and 0.9622 to 0.9493, respectively. When the SNR was 20 dB, the RMSE and CC of the proposed method were 0.0657 and 0.8831, respectively. Compared with the CNN, the RMSE and CC of CAE-CNN at 20 dB are optimized by 21.41% and 10.22%, respectively. The above results mean that the CAE-CNN method has good performance in the noise experiments.

## 5. Discussion

The experimental process and results of this paper are discussed as follows.

The reconstruction results are shown in Figure 10, and the evaluation index results are shown in Figure 11 and Table 5. From Figure 10, traditional algorithms can distinguish between arteries and veins, but cannot show the details of arterial stenosis. Traditional methods for solving underdetermined problems generally have two ideas. One is to treat it as a linear programming problem. An appropriate iterative algorithm is used to solve the optimal solution of the equation in the sense of least squares. Another is the singular value decomposition of the weight matrix. The reciprocal of the singular value of the weight matrix is used to construct its generalized inverse matrix, and it is multiplied by the potential difference to directly solve the blood flow velocity. The local optimal solution is obtained by these methods, but they do not fundamentally overcome the underdetermination and ill-posedness of the arterial blood flow profile reconstruction based on the electromagnetic effect. Therefore, the traditional algorithm cannot meet the needs of clinical diagnosis in imaging resolution. On the other hand, the adopted carotid artery stenosis model has strong nonlinearity. This leads to a better performance of traditional algorithms on simple models [36], but not in our work. A deep neural network can automatically learn and directly approximate the complex nonlinear mapping relationship between the input and output. It makes up for some of the shortcomings of traditional algorithms in solving nonlinear ill-posed problems.

In the neural network reconstruction algorithm, the reconstruction results of BP and CNN networks have some artifacts. The proposed method improves the imaging artifacts. Additionally, the results in Figure 11 and Table 5 show that the proposed method is more accurate in predicting the distribution of blood flow velocity. The reason why the reconstruction effects of BP and CNN are not as good as CAE-CNN is that the amount of reconstructed information is insufficient. Only 11 potential difference data are used as the input of BP and CNN. CAE has the ability to obtain effective low-dimensional representation of input by reconstructing input data and has achieved good results in [37,38,39,40]. CAE extracted the low-dimensional features of the weight matrix and obtained the details of the reconstruction information. After data domain transformation, the dimension of reconstruction information data is expanded from 11 to 11×60. In this way, the reconstruction information for the CAE-CNN network is increased and more detailed. The network structure and parameters of CNN in the comparison method is the same as that in the proposed method. The results of CAE-CNN are better than those of CNN, indicating that a reconstruction information increase is effective for improving the imaging quality. However, there are losses in the training process of CAE, so that the extracted weight matrix features cannot contain all the weight information. This is one of the reasons for the loss during the final blood flow velocity reconstruction. Figure 12, Figure 13 and Figure 14 show the results of noise resistance tests by each method. The reconstruction performance of CAE-CNN in noise test is better than other comparison methods. As shown in Figure 14, when SNR changes from 30 to 20 dB, the evaluation index largely deteriorates. This phenomenon can be attributed to the problem that the noise signal is amplified invisibly due to the enlargement of reconstruction information. However, when SNR = 20 dB, the reconstruction effect of the CAE-CNN method is still better than that of other comparison methods. Furthermore, the flow-induced potentials generated by the carotid artery will be extremely small. They will be contaminated by large bio-potentials (such as electrocardiosignal) lying in the same frequency range as the flow-induced signals. The effects of such bio-potentials may well be harder to eliminate than the Gaussian white noise signals. The proposed method is not able to be tested for reconstruction performance in this noisy environment due to the lack of clinical data.

For the effectiveness of the proposed method in clinical workflow, the authenticity of the generated data sets is very important. A carotid artery model with various stenoses was developed based on anatomical knowledge. The establishment of the model is considered in three aspects: the complexity of the model structure, the setting of conductivity and the setting of blood flow velocity. The structure and size of the model were designed according to the real cross-section of the human neck fascial layers. The area of real neck vessels is very small, and the change of the potential difference signal caused by vascular lesions is weak. Without considering the true location and size of the blood vessels on the whole cervical cross-section, the imaging effect of the trained network will be reduced in practical applications. Moreover, the model retains the main components of the neck cross-section, and the electrical conductivity of each part is set according to the real physiological parameters. This makes the conductivity distribution in the model more complex, enhances the nonlinearity, and increases the difficulty of reconstruction. In terms of velocity setting, the velocity of the stenosis is different from that of the normal vessels. So, the relationship between the stenosis rate and the blood flow velocity at the stenosis was analyzed. When the effect of stenosis rate on blood flow velocity is taken into account, the variation of the potential difference signal will be smaller than that when this effect is not taken into account. Although this requires a higher level of performance of the reconstruction algorithm for solving blood flow distribution, it is closer to the actual situation. It is noteworthy that there are other more complex stenosis conditions in the design of the distribution of arterial stenosis except the case mentioned in this paper. Additionally, the shape of the neck cross-section and the locations of the blood vessels were different between patients. Even though the blood flow velocity of the carotid vein fluctuates smoothly and evenly, it is still variable. The measured induced potential will also be affected by these changes. These more complicating factors are not considered in the proposed model. Additionally, the established simple vessel model does not cover the relationship between blood flow velocity and the stenosis rate corresponding to all stenosis lesions, and there are some idealized factors.

## 6. Conclusions

According to the electromagnetic effects of blood flow, a DNN method is proposed to reconstruct the arterial blood flow profile in this paper. The framework of the proposed method is established and trained by the data set obtained from a 2D carotid artery stenosis model. The potential difference measured by the electrodes distributed on the neck skin is used as reconstruction information, and the blood velocity distribution is regarded as a reconstruction target for obtaining the arterial blood flow profile image. Different from the previous methods, the proposed method firstly increased the dimensions of limited reconstruction information data, and then it predicted the blood flow velocity distribution. The CAE network is trained by the input weight matrix to extract weight matrix features. The weight matrix features are combined with the potential difference on the basis of the specific mathematical relationship for calculating the blood velocity distribution features. After the above process, the reconstruction information is increased in dimension and supplemented with details. Then, the blood velocity distribution features are divided into training sets and testing sets for CNN. Additionally, the testing sets are input into the trained CNN to predict the blood flow velocity and reconstruct high-quality arterial blood flow profile images. The reconstruction results of 817 samples that have not been used for training show that the RMSE and CC of the proposed method were 0.0333 and 0.9721, respectively. Additionally, the performance of the proposed method is better than Tikhonov, BP and CNN, both in noiseless and noisy tests.

In the future, we plan to improve the network structure to make it more robust to noise. On the side, in the process of modeling, more complex arterial stenosis conditions and the corresponding relationship between blood flow velocity and stenosis rate should be considered to improve the generalization ability of the proposed method.

## Figures and Tables

**Table 1:** The specific structure and parameters of the CAE encoder.

Layer	Type of the Layer	Kernel	Filter	Padding	Output Size
1	Conv (Tanh)	1 × 602	2	valid	11 × 2000 × 2
2	Conv (Tanh)	1 × 1001	4	valid	11 × 1000 × 4
3	Max-pooling	1 × 201	–	–	11 × 800 × 4
4	Conv (Tanh)	1 × 401	8	valid	11 × 400 × 8
5	Conv (Tanh)	1 × 201	16	valid	11 × 200 × 16
6	Max-pooling	1 × 101	–	–	11 × 100 × 16
7	Conv (Tanh)	1 × 51	32	valid	11 × 50 × 32
8	Conv (Tanh)	1 × 26	60	valid	11 × 25 × 60
9	Max-pooling	1 × 15	–	–	11 × 11 × 60

**Table 2:** The specific structure and parameters of the CAE decoder.

Layer	Type of the Layer	Kernel	Filter	Padding	Output Size
1	Bilinear interpolation	–	–	–	11 × 25 × 60
2	Deconv (Tanh)	1 × 26	60	same	11 × 50 × 60
3	Deconv (Tanh)	1 × 51	32	same	11 × 100 × 32
4	Bilinear interpolation	–	–	–	11 × 200 × 32
5	Deconv (Tanh)	1 × 201	16	valid	11 × 400 × 16
6	Deconv (Tanh)	1 × 401	8	valid	11 × 800 × 8
7	Bilinear interpolation	–	–	–	11 × 1000 × 8
8	Deconv (Tanh)	1 × 1001	4	valid	11 × 2000 × 4
9	Deconv (Tanh)	1 × 602	2	valid	11 × 2601 × 2
10	Conv (Tanh)	1 × 1	1	same	11 × 2601 × 1

**Table 3:** The specific structure and parameters of the CNN.

Layer	Type of the Layer	Kernel	Filter	Padding	Output Size
1	Conv + BN + Sigmoid	1 × 31	6	valid	11 × 30 × 6
2	Max-pooling	1 × 16	–	–	11 × 15 × 6
3	Conv + BN + Sigmoid	1 × 1	12	valid	11 × 15 × 12
4	Max-pooling	1 × 9	–	–	11 × 7 × 12
5	Conv + BN + ReLU	1 × 1	24	valid	11 × 7 × 24
6	Max-pooling	1 × 2	–	–	11 × 6 × 24
7	Reshape	–	–	–	1 × 1 × 1584
8	FC 1 + ReLU	–	–	–	1 × 1 × 2601
9	Dropout	–	–	–	–
10	FC 2	–	–	–	1 × 1 × 2601

**Table 4:** Typical value of electrical conductivity of human neck tissue.

Biological Tissue	Conductivity (S/m)	Biological Tissue	Conductivity (S/m)
skin	0.005	fat	0.1
muscle	0.2	esophagus	0
cervical spine	0.001	glands	0.6
trachea	0	artery	1.12
vein	1.12		

**Table 5:** The average results of evaluation indexes for 817 noiseless samples.

	Method	TK	BP	CNN	CAE-CNN
Indicators					
RMSE		0.1330	0.0906	0.0770	0.0333
CC		0.4366	0.7663	0.8358	0.9721

## Data Availability

Not applicable.
